# Dynamics of SARS-CoV-2 Variants of Concern in Brazil, Early 2021

**DOI:** 10.3389/fpubh.2021.784300

**Published:** 2021-12-23

**Authors:** José Eduardo Levi, Cristina Mendes Oliveira, Bianca Della Croce, Paulo Telles, Annelise Correa Wengerkievicz Lopes, Camila Malta Romano, Diego Bezerra Lira, Anna Claudia Mello de Resende, Flávia Paiva Lopes, André Arroyo Ruiz, Gustavo Campana

**Affiliations:** ^1^Dasa, São Paulo, Brazil; ^2^Tropical Medicine Institute, Faculdade de Medicina da Universidade de São Paulo, São Paulo, Brazil; ^3^Department of Epidemiology and Biostatistics, Universidade Federal Fluminense, Rio de Janeiro, Brazil; ^4^Hospital das Clinicas HCFMUSP, Faculdade de Medicina, Universidade de São Paulo, São Paulo, Brazil

**Keywords:** SARS-CoV-2, variants of concern, alpha, gamma, reinfections

## Abstract

Brazil is the country with the second-largest number of deaths due to the coronavirus disease-2019 (COVID-19). Two variants of concern (VOCs), Alpha (B.1.1.7) and Gamma (P.1), were first detected in December 2020. While Alpha expanded within an expected rate in January and February 2021, its prevalence among new severe acute respiratory syndrome coronavirus 2 (SARS-CoV-2) cases started to decrease in March, which coincided with the explosion of Gamma variant incidence all over the country, being responsible for more than 95% of the new cases over the following months. A significantly higher viral load [i.e., mean cycle threshold (Ct) values] for Gamma in comparison to non-VOC samples was verified by the analysis of a large data set of routine reverse transcription–PCR (RT–PCR) exams. Moreover, the rate of reinfections greatly increased from March 2021 onward, reinforcing the enhanced ability of Gamma to escape the immune response. It is difficult to predict the outcomes of competition between variants since local factors like frequency of introduction and vaccine coverage play a key role. Genomic surveillance is of uttermost importance for the mitigation of the pandemic.

## Introduction

Since the beginning of the SARS-CoV-2 epidemic in December 2019, this beta-coronavirus has been diversifying at an average rate of 1–2 nucleotides per month, generating hundreds of variants worldwide. Mutations conferring adaptive advantage to a strain result in its geographical and quantitative expansion at the expense of other variants/lineages.

This became evident by November/December 2020, when three variants emerged independently in distant locations such as the United Kingdom (UK) (1), South Africa (2), and Brazil (3). These three variants rapidly became the predominant SARS-CoV-2 isolates in the regions where they surged, thus, being named variants of concern (VOCs) to distinguish them from other variants and to highlight the need for intensified surveillance. VOCs have a number of spike gene mutations, some in the receptor-binding domain (RBD). Among RBD mutations, these variants share N501Y, as previously postulated by *in vitro* experiments, that increases the affinity of the S-protein RBD to the cellular receptor ACE2 (4), thus, facilitating cell invasion.

Variant B.1.1.7, named Alpha, was recognized as a VOC in December 2020, when UK authorities made it public knowledge that this variant was increasingly prevalent in some parts of the country, despite an overall decrease in the number of new cases, suggesting enhanced transmissibility (1). This led to restrictions in dozens of countries on flights and travelers from the UK. The discovery and surveillance of B.1.1.7 were eased by an unpredicted reverse transcription–PCR (RT–PCR) anomalous result, named “S-dropout” (5), which is observed when carriers of B.1.1.7 are submitted to an RT-PCR method using the ThermoFisher COVID-19 TaqMan assay. This assay has three SARS-CoV-2 genomic targets: nucleocapsid (N), ORF1ab, and spike (S). Unexpectedly, the assay was able to amplify two targets (N and ORF1ab) but failed to detect the S gene. Upon sequencing, it was shown that B.1.1.7 carries 23 mutations, which included two deletions and six non-synonymous mutations in the S gene (6). One of the deletions (Δ21765–21770/HV 69–70) abrogates a primer (or probe) binding site, thus leading to S-gene target failure (SGTF). Although other variants, like B.1.375, also carry this deletion, in a scenario of B.1.1.7 expansion, SGTF represents B.1.1.7 infections and is used as a trusted proxy for it (5).

As the ThermoFisher COVID-19 TaqMan assay is currently adopted in many UK laboratories, epidemiological data from B.1.1.7 are much more abundant than from the other two VOCs, which were initially recognized and investigated by RNA sequencing, a cumbersome and expensive method that provides a lower throughput. B.1.1.7 is associated with significantly higher viral loads (7), which also impacted the rate of transmission. By collecting data from hundreds of thousands of patients, UK scientists showed that B.1.1.7 is also more lethal than non-B.1.1.7 variants (8), although current SARS-CoV-2 vaccines and monoclonal antibody therapies appear to be effective against this variant.

Dasa is the largest clinical pathology laboratory in South America, having performed ~4.5 million COVID-19 RT-PCRs since February 2020. Due to the constant shortage of reagents and high-throughput demand, several platforms and kits are used in its laboratories. In December 2020, a routine saliva-based RT-PCR test was introduced, employing, by chance, the same ThermoFisher reagent, that led to the detection of the first two cases of B.1.1.7 in Latin America (9). The use of the ThermoFisher multiplex assay was further expanded to nasopharyngeal swabs, allowing for the accumulation of a large dataset of B.1.1.7 cases.

In January 2021, P.1 (Gamma) emerged and caused a tragedy in the Amazonas state. Its increased transmissibility was evidenced by the short period between P.1 emergence and it being found in virtually 100% of the cases in that region (10). P.1 also carries N501Y and shares with B.1.351, the receptor-binding domain (RBD) E484K mutation, associated with immune evasion, which is one of the explanations for the explosion of cases in the region previously reported to present, at that time, probably, the highest seroprevalence in the world, of about 66% (11). Later on, the E484K mutation also emerged independently among Alpha isolates, although it never became prevalent worldwide (GISAID).

It is revealing that the first Amazon P.1 autochthonous case was described in a well-documented re-infection episode (12), and it has been estimated that 28% of the second wave cases in Amazonas state may have been re-infections (13). The aim of this study is to report the dynamics of these two SARS-CoV-2 VOCs, Gamma, and Alpha, during the first trimester of 2021, in Brazil.

## Methods

### Samples

Combined naso/oropharyngeal swabs were collected between January and March 2021 from subjects seeking one of the 800 Dasa units, which are spread all over the country, for routine SARS-CoV-2 RT-PCR testing. This population presented the full range of the clinical spectrum, from severely ill hospitalized patients to asymptomatic travelers. Swabs were dipped in 3 ml of sterile saline and transported under refrigeration (2–8°C) to the central laboratory located in Barueri, São Paulo state, Brazil.

### RT–PCR and VOC Identification

Samples were processed in no more than 72 h. An aliquot of 300 μl was submitted to RNA extraction in a platform that integrates an automated pipettor (Janus; Perkin-Elmer, São Paulo, Brazil) to a nucleic acid extraction system (Chemagic 360; Perkin-Elmer, São Paulo, Brazil) employing Chemagic Viral 300 (Perkin-Elmer, São Paulo, Brazil) reagents. Ten microliters of the eluate was added to the ThermoFisher COVID-19 TaqMan (ThermoFisher, São Paulo, Brazil) assay reagents according to the manufacturer's instructions. All amplification curves were inspected visually, and data were electronically transferred to the central laboratory information system. Ct values were stored in the database but not included in the final report to patients and prescribers. Samples presenting an N gene Ct value below 30, in addition to ORF 1ab amplification, but no S gene Ct value was assigned as B.1.1.7. The Ct value for the N gene was taken as a surrogate of the viral load.

### P.1 Assignment

A randomly selected subset of positive SARS-CoV-2 RNA samples with N gene Ct value below 30, but showing S gene amplification, were submitted to two VOC-identifying assays (14, 15). These assays are unable to distinguish P.1 from B.1.351. Those harboring the nine-nucleotide deletion in the ORF1ab (NSP6) common to the three VOCs were categorized as P.1, while those lacking the deletion were assigned as “other lineages.” Since during the study period there was no report of any B.1.351 isolate identified in Brazil by other surveillance programs, samples with this profile were considered as P.1.

### Reinfection Analysis

For the investigation of reinfections, the full dataset (3,369,718 entries/samples) was used, including samples collected since March 2020. Patients with negative results were removed from the base. Patients with more than one test in a single day had positive results prioritized. Reinfection was defined as a second positive RT-PCR test, with a minimum window of 120 days between positive tests. The resulting database was filtered to account for reinfections that occurred after the window of 120 (4), 150 (5), and 180 days (6 months).

## Results

**Figure 1:**
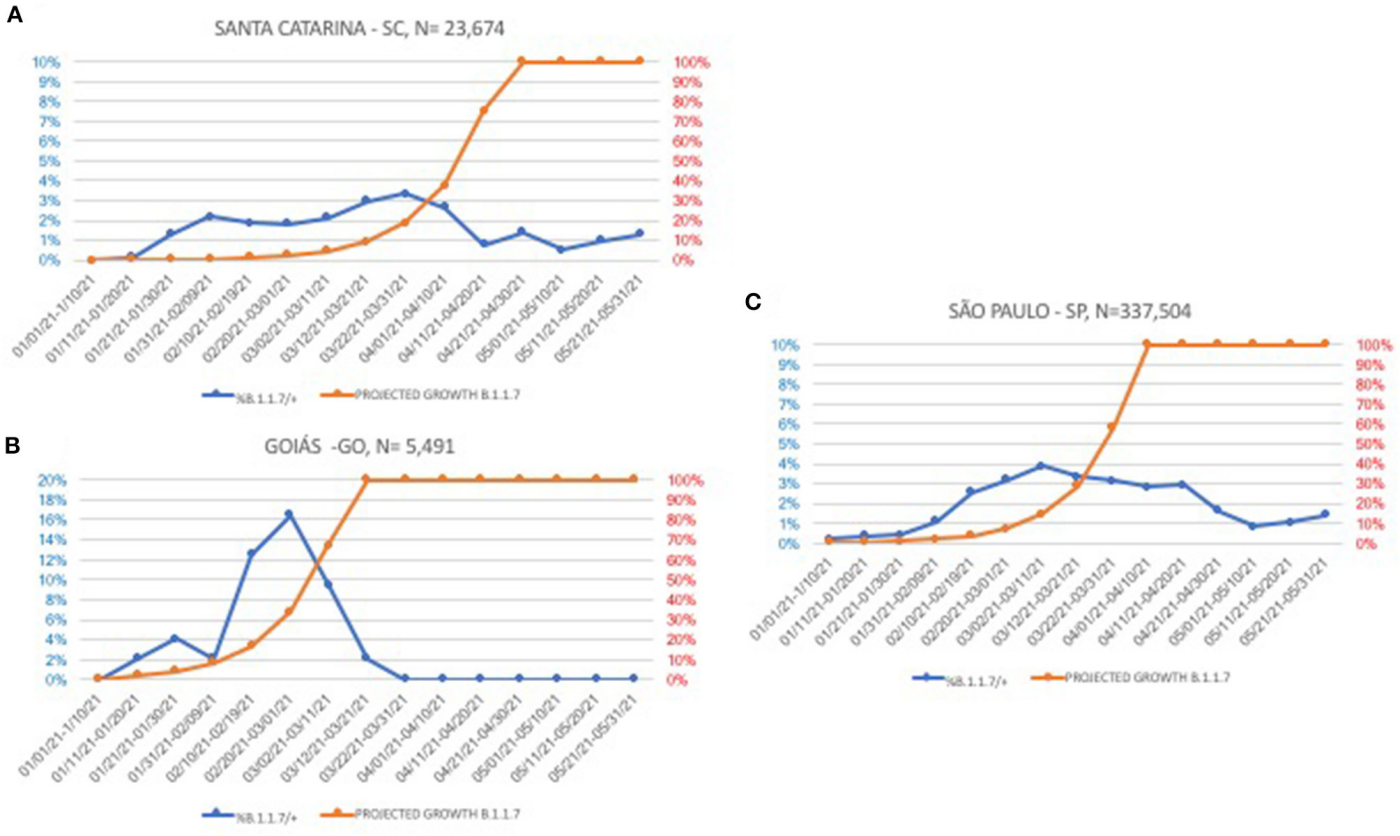
Temporal evolution of B.1.1.7 (S-gene target failure, SGTF) in three Brazilian states with more than 5,000 samples tested. Blue lines, actual frequencies, orange lines, projections with a 10-day doubling time fixed rate. **(A)** Santa Catarina state; **(B)** Goiás state; and **(C)** São Paulo state.

From January 1 to March 31 2021, a total of 361,198 nasopharyngeal swabs were submitted to the Thermo assay, with 109,349 (30.27%) testing positive. Within the positive group, 89,165 (81.5%) samples displayed an N-gene Ct value ≤ 30 and, among these, 1,891 (2.1% [1,891/89,165] of the cases with low N gene Ct and 0.5% [1,891/361,198] of the total) showed no S-gene amplification. São Paulo state has the larger dataset; thus, it is where the majority of the SGTFs (1,527; 80.7%) were identified. At least one sample from each of the 25 out of the 27 Brazilian states (exceptions were Amapá and Roraima) was tested, and SGTF was present in 17 states. Three states with more than 5,000 samples analyzed were selected for a detailed temporal analysis (Figure 1). In this analysis, real data are shown along with a projected growth rate, adopting a B.1.1.7 doubling frequency every 10 days (16).

The prevalence of P.1 verified in five Brazilian states is shown in Table 1. A gradient of P.1 frequencies is observed, being higher in the South/Southeast and lower in Northeast and Central regions. For a dynamic perspective, where available, frequencies are presented on a monthly basis (Tables 2, 3).

**Table 1:** Prevalence of P.1 severe acute respiratory syndrome coronavirus 2 (SARS-CoV-2) ribonucleic acid (RNA)-reactive samples in five Brazilian states.

**State/Region**	**Number of** **samples**	**Prevalence** **(95% CI)**	**Time period**
Brasília (Central)	87	15% (8.9–23.9)	Jan–Mar, 2021
Maranhão# (Northeast)	182	60% (52.6–66.7)	Feb-21
Santa Catarina (South)	200	78% (71.7–83.2)	Feb–Mar, 2021
São Paulo* (Southeast)	180	80% (73.6–85.2)	1^st^ week Mar, 2021
Rio de Janeiro (Southeast)	157	84% (77.5–90.0)	Jan-Mar, 2021

*#All samples are from the state capital São Luis.*

*All samples are from São Paulo metropolitan area.

*Means and confidence intervals (CIs) are shown*.

**Table 2:** Frequency of P.1 and non-P.1 variants in SARS-CoV-2 RNA-reactive samples over time in Rio de Janeiro state.

**Rio de Janeiro**				
	**P.1**	**Non-P.1**	**Total**	P.1% (95% CI)*
February 15^th^-27^th^, 2021	38	15	53	71.7 (58.4–82.0)
March 1^st^-6^th^, 2021	41	5	46	89.1 (77.0–95.3)
March 21^st^-31^st^, 2021	46	1	47	97.9 (88.9–99.6)
Total	125	21	146	85.6 (79.0–90.4)

*Z-test, performed with R software (17).

**Table 3:** Frequency of P.1 and non-P.1 variants in SARS-CoV-2 RNA-reactive samples over time in Brasília, national capital.

**Bras**í**lia – DF**				
**YEAR 2021**	**P.1**	**Non-P.1**	**TOTAL**	P.1% (95% CI)*
January	1	37	38	2.6 (0.1–15.4)
February	3	27	30	10.0 (2.6–27.7)
March	8	2	10	80.0 (44.2–96.4)
Total	12	66	78	15.4 (8.5–25.7)

*Performed with R software with Yates' continuity correction.

Cycle threshold (Ct) values from samples carrying either P.1, B.1.1.7, or other variants, obtained in the same period, were compared (Table 4) from the samples. Only N gene data are presented, but ORF1ab Ct values are very similar and reflected differences of the same magnitude (data not shown).

**Table 4:** Nucleocapsid (N) gene cycle threshold (Ct) values according to SARS-CoV-2 variants.

**Gene N Ct values**					
	**N**	**MEAN**	**SD (±)**	**dif. in means (** ***p*** -value)*	**Median**
P.1	222	18.52	4.51	−2.14 (*p* <0.001)#	17.65
B.1.1.7	1,893	19.86	4.73	−0.80 (*p* = 0.06)	19.08
Other	114	20.66	4.27	Ref.	20.04

*Welch's modified two-sample t-test.

*^#^Difference in means was statistically significant*.

**Figure 2:**
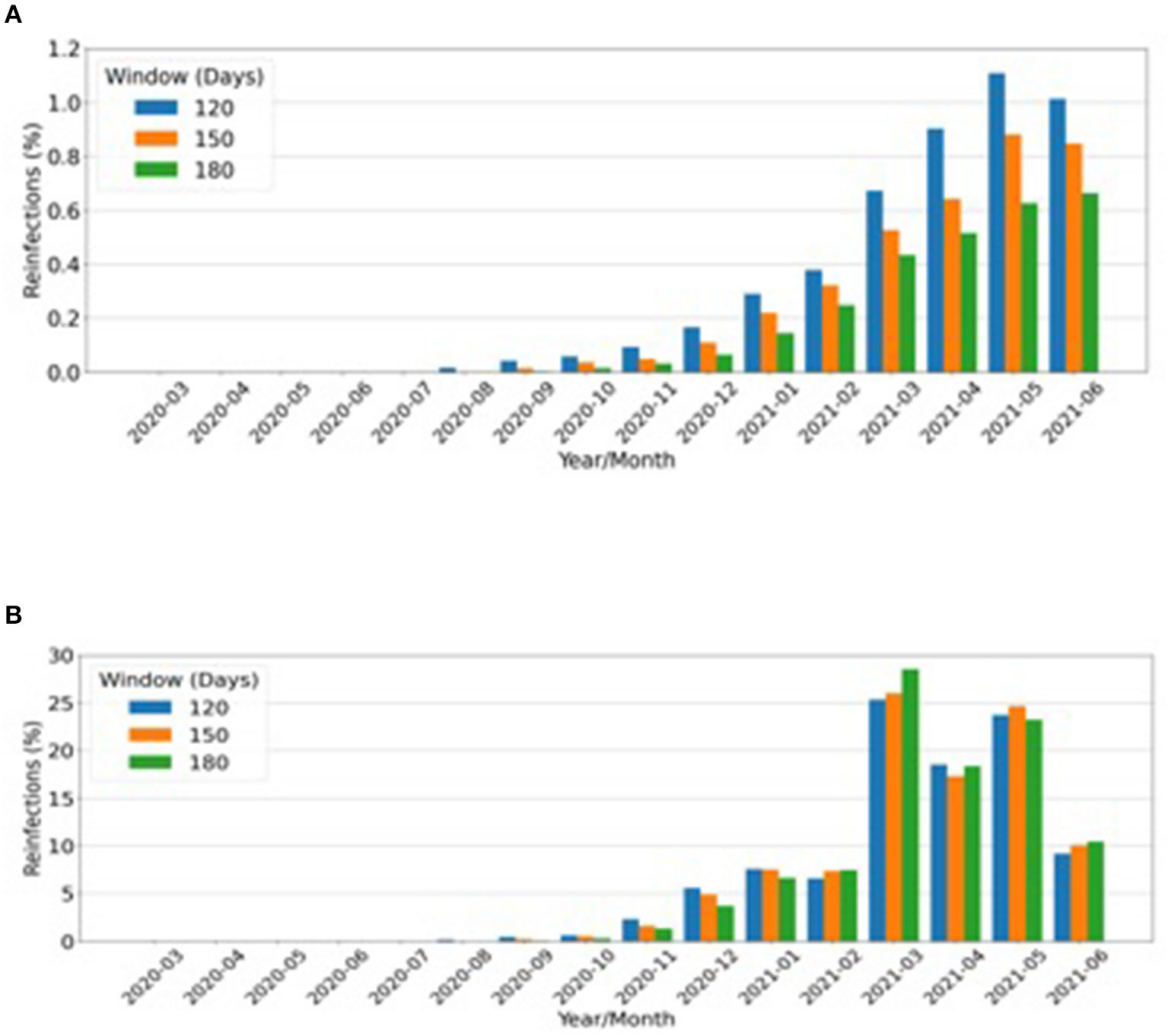
Putative reinfections considering all positive real-time (RT)-polymerase chain reactions (PCRs). **(A)** Monthly rate over a total number of reinfections from March 2020 to June 2021. **(B)** Absolute number of reinfections that occurred after the periods of: blue bars, 120 days (4 months); orange bars, 150 days (5 months); green bars, 180 days (6 months).

A total of 3,430, 2,607, and 1,964 reinfections were observed in 120, 150, or 180 days between two positive RT-PCRs, respectively. These reinfections represented ~1% of all RT-PCR reactive samples in the months corresponding to the peak of the second wave, as shown in Figure 2A. March 2021 accounted for at least one-quarter of all reinfections of the evaluated period (March 2020 to June 2021) (Figure 2B).

## Discussion

During the first months of the pandemic, SARS-CoV-2 variants were identified and used as tools for tracing transmission routes and international movement. Many studies investigated whether viral mutations/variants could justify the diversity of clinical outcomes in infected patients with similar demographic backgrounds and health conditions. However, except for the current universal mutation D614G, which affects the spike protein gene and is related to increased transmission (18), no other significant associations between viral genotype and phenotypic expression could be made.

From its emergence in late December 2020 (9), the tracking of B.1.1.7 in January 2021 and the first weeks of February 2021 revealed a doubling rate of ~10 days, as predicted and further verified in the United States (19). Surprisingly, in late February, its incidence rate became flat. At that moment, an increase in the number of new cases, recognized as a COVID-19 “second wave,” was being reported all over Brazil. Samples randomly selected from some capitals revealed the dominance of the P.1 VOC. It was inevitable to conclude that P.1 clearly prevailed over B.1.1.7 and P.2, which was the major lineage in December 2020 all over the country (20). However, it must be acknowledged that assignment to P.1 was solely based on the NSP6 deletion common to the three VOCs described at the time, Alpha, Beta, and Gamma. While Alpha was distinguished based on the failure to amplify the S gene fragment, the Beta variant was dismissed based on the absence of this VOC in Brazil at the time and later on. Another limitation of the study is the definition of re-infection, which relies on two consecutive positive RT-PCR exams with a time interval longer than 4 months. Although the majority of COVID-19 cases have a short course of about 2 weeks, viral reactivation cannot be excluded. Reinfection is categorically determined by sequencing both isolates and showing that they undoubtedly belong to distinct SARS-CoV-2 lineages, which was not performed here.

It has been reported that both B.1.1.7 (7) and P.1 (2, 10) harbor, on average, higher viral loads when compared to other coexisting variants. The median N gene Ct value reported here from P.1 isolates (17.65) is 2.5 log_10_ lower than that of other lineages, resembling the difference observed in the Amazonas state by Naveca et al., between P.1 and non-P.1 isolates (10). This 2.5 log_10_ difference between median values corresponds to P.1 viral loads 245 and 27 times higher than those of other variants and B.1.1.7, respectively, which certainly contribute to its enhanced transmissibility. Even though a lower mean Ct value for B.1.1.7 compared to non-B.1.1.7 viruses were observed, the difference was not as significant as verified in the UK (21).

Dissemination of P.1 in a population with high SARS-CoV-2 seroprevalence, such as the Amazonic, was possible in part because of S-gene mutations that allowed immune escape (10, 12, 13). A large number of reinfections are implicit according to this rationale, and indeed it has been calculated that 16.9–31% of the infections in the city of Manaus, Amazonas between January and March 2021 were due to reinfection by P.1 (22). Apparently, our estimate of up to 1% reinfection rate contrasts with the 16.9–31% Amazonas reinfection estimate, but these cannot be compared, since our denominator is the total number of positive RT–PCRs from different regions of the country. If the data are restricted to two RT–PCRs in the period, a much higher estimate will be found. However, this approach is susceptible to enormous bias; hence, we adopted a calculation method that offered, perhaps, a better illustration of the magnitude of reinfections for the population of the whole country.

Competition between variants for the niche of susceptible subjects is expected, and the outcome is difficult to predict. India experienced a similar situation, where B.1.1.7 was the dominant VOC until the emergence of B.1.617, which, since July 2021, has been responsible for the overwhelming majority of new cases worldwide^1^.

It is tempting to attribute the predominance of one VOC over another to the mutational pattern, reflecting higher infectivity/transmission properties. Nevertheless, epidemiological factors do play an important role. For instance, the number of P.1-infected subjects leaving from Amazonas state to other Brazilian regions in December 2020, principally going to São Paulo city, was certainly much higher than that of travelers coming to São Paulo from the UK and other regions where B.1.1.7 was prevalent. Thus, it is inadvisable to compare the replacement of B.1.1.7 by B.1.617.2 in India, where B.1.1.7 represented 26% of the new cases in early March and <1% in early June^1^, since B.1.1.7 never surpassed the 5% frequency in Brazil.

This scenario suggests that while surveillance of travelers and frontiers is necessary to avoid the introduction of new VOCs, there is a considerable risk of further mutations in the P.1 lineage that could possibly lead to even more transmissible and pathogenic strains that may also threaten the efficacy of current vaccines. Intensifying real-time national genomic surveillance of new cases, such as fully vaccinated individuals presenting with severe COVID-19 disease, will be of paramount importance in fighting the pandemic in this heavily affected country.

## Data Availability Statement

The raw data supporting the conclusions of this article will be made available by the authors, without undue reservation.

## Ethics Statement

The studies involving human participants were reviewed and approved by Comitê de Ética em Pesquisa do Hospital Nove de Julho, São Paulo, Brazil. Approval: CAAE 51655621.0.0000.5455. The Ethics Committee waived the requirement of written informed consent for participation.

## Author Contributions

JL conceived the study, analyzed the data, and wrote the manuscript. CO, BC, and CR performed the variant-definition experiments, analyzed the data, and reviewed the manuscript. PT performed the statistical analysis and reviewed the manuscript. AL assisted in sample selection, analyzed the data, and reviewed the manuscript. DL, ARe, and ARu extracted, prepared and analyzed the data and reviewed the manuscript. FL conceived the study and reviewed the manuscript. GC analyzed the data and reviewed the manuscript. All authors contributed to the article and approved the submitted version.

## Funding

This study was funded by Dasa.

## Conflict of Interest

The authors declare that the research was conducted in the absence of any commercial or financial relationships that could be construed as a potential conflict of interest.

## Publisher's Note

All claims expressed in this article are solely those of the authors and do not necessarily represent those of their affiliated organizations, or those of the publisher, the editors and the reviewers. Any product that may be evaluated in this article, or claim that may be made by its manufacturer, is not guaranteed or endorsed by the publisher.

## Footnotes

^1^Available online at: https://covariants.org/per-country (accessed November 15, 2021).
